# Three-dimensional (3D) polymer—metal–carbon framework for efficient removal of chemical and biological contaminants

**DOI:** 10.1038/s41598-021-86661-w

**Published:** 2021-04-08

**Authors:** V Sasidharan, Deepa Sachan, Divya Chauhan, Neetu Talreja, Mohammad Ashfaq

**Affiliations:** 1 School of Life Science, BS Abdur Rahman Crescent Institute of Science and Technology, Chennai, India; 2 Center for the Environment, Indian Institute of Technology Guwahati, Guwahati, 781039 India; 3 Department of Chemical and Biomedical Engineering, University of South Florida, Tampa, USA; 4 Multidisciplinary Research Institute for Science and Technology, IIMCT, University of La Serena, 1015 Juan Cisternas St., La Serena, Chile

**Keywords:** Materials science, Nanoscience and technology

## Abstract

The continuously increased existence of contaminants such as chemical and biological mainly dye, bacteria, and heavy metals ions (HMI) in water bodies has increased environmental concern due to their hostile effects on living things. Therefore, there is necessity to be developed newer materials that skirmishes such environmental menace. The present works focus on the synthesis of a novel three-dimensional (3D) polymer-metal–carbon (3D-PMC) framework for the exclusion of contaminants (chemical and biological) from water bodies. Initially, polyurethane (PU) foam was treated with nitric acid and used as a framework for the development of 3D-PMC materials. The copper nanosheet (Cu-NS) was deposited onto the functionalized PU foam to produce Cu-NS-PU material. The mechanically exfoliated graphene was mixed with chitosan to produce a graphene-chitosan homogenous suspension. The produce homogenous suspension was deposited Cu-NS-PU for the development of the 3D-PMC framework. The prepared 3D-PMC framework was characterized by scanning electron microscopy (SEM), Energy Dispersive X-Ray Analysis (EDX), Fourier-transform infrared spectroscopy (FT-IR), and X-rays diffraction (XRD) analysis. The prepared 3D-PMC framework was subjected to various adsorption parameters to assess the sorption ability of the material. The prepared 3D-PMC framework was effectively used for the removal of chromium (Cr) metal ions and Congo-red (CR) dye from the water system. The synthesis of the 3D-PMC framework is simple, novel, cost-effective, and economically viable. Therefore, the prepared 3D-PMC framework has the potential to be used as a filter assembly in water treatment technologies.

## Introduction

The incessant increasing occurrence of biological and chemical pollutants such as heavy metal ions (HMI), dyes, and biological contamination (bacteria and fungi) in the water system increased the environmental menace globally. Contamination of water bodies by toxic HMI such as chromium (Cr), arsenic (As), lead (Pb), cadmium (Cd), and mercury (Hg) is one of the solemn threats globally due to their high toxicity to living being including human health. The existence of pollutants in water bodies has increased environmental worry worldwide due to its adverse effects on animals, plants as well as human health^1–8^.

Textile industrial waste mainly contains various pollutants like alkalis, inorganic and organic salts, dye, and HMI. Cr(VI) is extensively used for the leather dyeing procedure. Moreover, high solubility in water makes it easier to enter the food chains, thereby higher accumulation within the body that leads to severe health issues. The incessant exposure of Cr(VI) to a human might because damaging various organs like the liver, kidney, and improper functioning of the circulatory and nervous system. Congo Red (CR) dye is a recognized pollutant and highly toxic and cancerous^9,10^. Gram-negative (Escherichia coli (*E. coli*)), and Gram-positive (Staphylococcus aureus (*S. aureus*)) bacterial strains some of the most prevalent bacteria in water. Biological contamination is one of the major issues nowadays that developed various health-related problems such as diarrhoea, cholera, and bacterial infectious disease^11–15^. In this context, treatment or confiscation of such contaminants (both chemical and biological) is necessary for pure drinking water.

Numerous processes mainly physical, chemical, and biological treatment has been used to treat all contaminants from water. However, these processes are not efficient for the confiscation of either chemical or biological pollutants from water bodies. Hence, there is a necessity to be developed newer competent, economically viable adsorbent materials or filters that might be eliminated chemical and biological contaminants from the water stream^16–19^. In this context, polymeric composite contains different surface functional groups that efficiently remove various contaminants from water.

Numerous polymers such as polyvinyl alcohol (PVA), chitosan, poly (methyl methacrylate) (PMA), poly-aniline, poly-pyrrole, poly-urea, poly-urethane have been used to a developed adsorbent or filter materials for the confiscation of a pollutant from water. However, high swelling ability and low affinity of adsorbent or filter materials towards contamination remain a concern^20–24^. In this context, hybrid materials or a combination of more than two or three materials might be overcome such associated issues with high removal efficiency.

Chitosan has considered necessary filtration characteristics. Moreover, Chitosan is a non-toxic, biocompatible, biodegradable polysaccharide, extraordinary adsorbing ability towards anionic dyes like CR-dye, and exceptional antibacterial ability, thereby extensively used in numerous filtration applications (water to air purification)^24–26^. Carbon-based nanomaterials such as CNTs, CNFs, graphene, and graphene oxide exhibit a promising role in the cleansing of pollutants from water bodies. Graphene is considered a futuristic material due to its exceptional characteristics like large specific surface area, and easily tunable properties according to the specific applications^27–31^. Therefore, graphene becomes a suitable candidate for the confiscation of contaminants from water.

Metal-NPs mainly Ag, Au, Zn, and Cu extensively used to treat biological pollutants like bacteria and fungi that inhibit the synthesis of protein, disruption of cells, and damaging DNA, thereby inhibit or kill microorganisms. The nano-sized Cu-NPs have the tremendous quality to clean out chemical as well as biological waste due to high reactivity^15,32,33^. The unique combination of all these materials makes a suitable candidate for the development of PMC based adsorbent or filter materials.

The proposed method of development of PMC framework-based filter or adsorbent is simple, novel, and economically viable, and efficiently removes both chemicals as well as biological contaminants from water. The prepared PMC scaffold was used for the confiscation of contaminants from water and proposed as the filter or adsorbent materials. The graphene and chitosan-coated on the PU-foams enhanced the efficiency of the filter or adsorbent materials towards both biological and chemical contaminants. Cu-NS deposited on the PU-foams enhances the antibacterial activity of the composite and formed a complex with HMI. Further, amide and carboxylate groups of chitosan of the composite facilitate adsorption of dye, heavy metal ions, and kill biological contaminants, thereby enhancing the sorption ability against both chemical and biological contaminants. The novelty is in the simplistic development of the low-cost polymer-metal–carbon-based materials as the efficient adsorbent materials or filters for the confiscation of both chemical and biological pollutants from water or purification of water. The different components of the adsorbent or filter material, namely, PU foam, Cu-NS, graphene, and chitosan, serve different purposes. The Cu-NS serves dual roles. (1) Facilitate the antibacterial activity or removal of biological contaminants, and (2) Facilitate the adsorption ability of Cr(VI) and CR-dye from water. PU foam serves dual roles. (1) Acts as the framework for the development of PMC materials, and (2) It provides interconnecting pores within the polymeric composite, thereby increasing the exposure of CU-NS and Chitosan to Cr(VI) and CR-dye. Chitosan serves triple roles. (1) It provides stability to the Cu-NS against leach out or rapid dissolution from PMC material, (2) Facilitate antibacterial activity or removal of biological contaminants from water, and (3) Enhance the sorption capacity of the Cr(VI) and CR-dye from water. Graphene enhances the sorption ability of the Cr(VI) and CR-dye from water. The unique combination of the polymer-transition metal–carbon composite dispersed with Cu-NS and graphene yielded the efficient adsorbent or filter material, used to remove both chemical and biological contaminants from water or provide pure water. The produced PMC materials were highly capable to remove Cr(VI), CR-dye, and bacterial contaminants from water. The method of synthesizing the PMC materials or polymer-transition metal–carbon-composite adsorbent or filter material in the present study is novel, facile, and economically viable.

## Materials and methods

### Materials

Chitosan, ammonia, acetic acid, graphite powder, polyurethane foam, nitric acid, chromium (VI), copper sulfate, Congo red (CR) dye, hydrochloric acid, sodium hydroxide, deionized (DI) water. All chemicals were used of high purity grade. All reagents were prepared in Milli-Q water.

### Preparation of PMC adsorbent materials

The synthesis of the Cu-NS deposited Pu-foam-graphene-chitosan-based framework or PMC filter or adsorbent material was started with chemical treatment of PU-foams using 1 M HNO_3_ to produce chemically-treated PU-foams. The produce chemically-treated PU-foams washed several times using DI water until the surface becomes neutral (pH ~ 7). Next, Cu-NS was synthesized by CBD process, for this 0.2 M CuSO_4_ was dissolved in 150 mL of DI water to produce a homogenous suspension. Approximately, 15 mL of NH_3_ solution was added in the homogenous suspension of CuSO_4_ to maintain the pH value of ~ 10–11. The homogenous suspension was kept in the water bath at 90 °C after 15 min treated PU-foam was dipped into the homogenous suspension for a different time interval (30–240 min) to synthesize PU-Cu-NS. The maximum deposition was observed at 180–240 min of Cu-NS onto the PU-foam. For dense growth or higher deposition of Cu-NS, we optimize 240 min of incubation time to prepare PU-Cu-NS samples. After deposition of Cu-NS onto the PU-foam, samples were dried at 80 °C for 24 h, and then samples were washed gently to remove excessive bonded Cu-NS onto the PU-foam. Next, washed PU-Cu-NS samples were dried at 80 °C for 24 h. The developed PU-Cu-NS samples were used for the preparation of PMC based adsorbent or filter materials.

**Figure 1:**
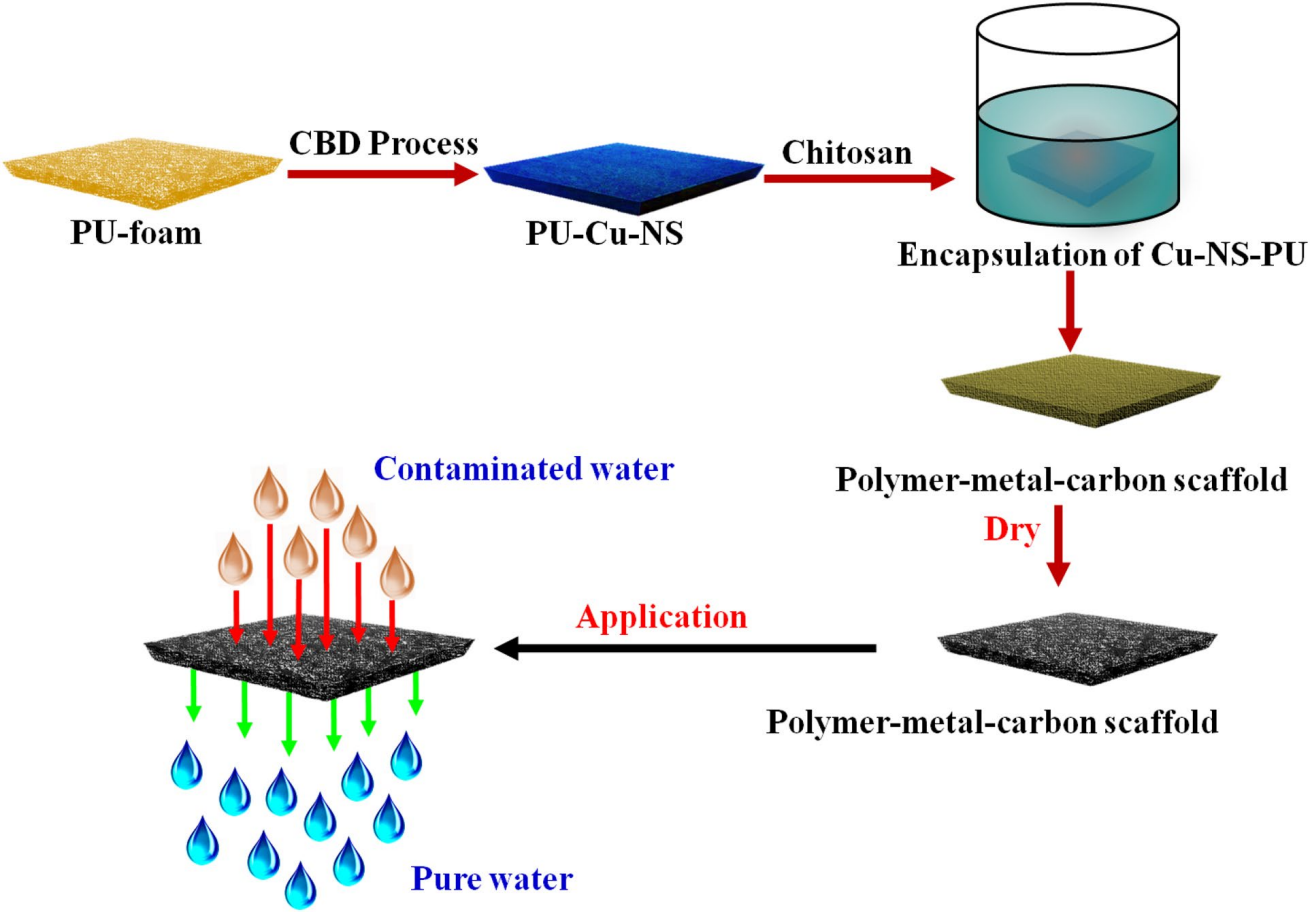
Schematic representation of the synthesis of the PMC framework as filter or adsorbent materials for environmental remediation application.

The graphene was synthesized from graphite powder using a mechanical exfoliation process^34^. Approximately 0.5–1.5 g of chitosan granules were dissolved in 120 mL of DI water with the help of continuous stirring and heating at 90 °C for 60 min. Next, 0.05% acetic acid was added into the chitosan solution to produce a homogenous solution of chitosan. Approximately, 0.1 to 0.5 g of mechanically exfoliated graphene was added into the homogenous solution of chitosan using continuous stirring at 300 rpm for 2 h at room temperature (~ 30 °C) to produce a chitosan-graphene homogenous solution. The produce chitosan-graphene homogenous solution used as an encapsulating agent for PU-Cu-NS samples by continuously stirring at 200 rpm for 2 h to produce PMC based adsorbents or filter materials. The developed 3D-PMC framework was used as a filter or adsorbent materials for the confiscation of all contaminants from water. Figure 1 shows a schematic illustration of the synthesis of a PMC framework used as the filter or adsorbent materials for environmental remediation application.

### Batch adsorption studies

A batch experiment was performed to analyze different influential factors such as time of adsorption, concentration, pH, and temperature over adsorption of Cr(VI) onto PMC framework material. Adsorption kinetics was studied at 150 mg L^−1^ of Cr(VI) and 10 mg L^−1^ CR using 10 mg PMC with different time intervals from 0–24 h at a constant speed of 100 rpm at 35 °C. pH study on Cr (VI) and CR using PMC was performed to analyze the better absorbance efficiency (mg L^−1^). Solution pH was adjusted by adding 1 N HCl and 1 N NaOH to maintain the pH range (2 to 9), while the adsorbent dose, temperature, and other parameters were kept constant. A stock solution of 1000 mg L^−1^ of Cr(VI) and CR was prepared. The test solutions of 25 mL volume having different adsorbate concentrations were prepared in conical flasks from the stock solution. A small amount (~ 0.01 g) of the prepared PMC was transferred to the conical flasks. The flasks containing the test solution and adsorbents were kept in a mechanical shaker (150 rpm) at room temperature (35 °C). The concentration of Cr(VI) in the solution was ascertained by using 1,5-diphenylcarbazide (DPC) method while CR was measured directly using a UV–VIS spectrophotometer. The wavelength of the detector was set at 540 nm and 496 nm, respectively. From the species balance equation, the amount of Cr (VI) and CR adsorbed by the prepared materials was calculated.

1 $$q=V(Ci-Ce)/W$$

where *q* is the loading (mg g^−1^) of Cr(VI) and CR and *Ci* and *Ce* are the initial and final (equilibrium) concentrations (mg L^−1^), respectively, of the solution. *V* is the volume (L) of the solution and *W* is the weight (g) of the adsorbent. All tests were done in triplicates to check the reproducibility.

### Antibacterial analysis

The antibacterial test analysis of the PMC based adsorbent materials was determined against both *E. Coli*, and *S. aureus*. The bacterial strains were cultured in a Luria Bertani broth (LB media) medium and incubate at 37 °C for 24 h in a bacterial incubator. Different doses (100, 200, 400, and 600 mg) of PMC based adsorbent materials were analyzed by using the plate count method. 1 mL of bacterial strains was mixed into 50 mL of phosphate buffer saline (PBS) solution with different amounts of PMC based adsorbent materials in a conical flask. The initial bacterial count was ~ 10^4^–10^5^ CFU mL^−1^. Approximately, 100 µL incubated bacterial samples were taken from each conical flask and spread over the LB agar medium plate and then incubated at 37 °C for 24 h. The bacterial culture without PMC based adsorbent materials served as the control for comparison purposes. All experiments were performed in triplicate to check reproducibility.

## Material characterization

The surface structure of the prepared PMC based adsorbent materials was characterized by using several characterization techniques such as field emission-scanning electron microscopy (FE-SEM), Energy dispersive X-rays (EDX), X-ray diffraction (XRD), and Fourier transform-infrared (FT-IR) spectroscopy. The surface texture of the prepared PMC based adsorbent materials was characterized by using FE-SEM (MIRA3-, TESCAN, A.S., Brno, Czech Republic). The presence of Cu-NS within the PMC based adsorbent materials was observed by using EDX analysis (Oxford, Inc., Germany). The crystalline pattern of the PMC based adsorbent materials was determined using XRD analysis with Cu Ka radiation (k = 1.54178 A°) at a scan rate of 5 °C per min. The surface functional group of the prepared PMC based adsorbent materials was ascertained by FTIR spectra with a wavelength range (400–4000 cm^−1^) (Brucker, Germany).

## Result and discussion

### SEM and EDX analysis

**Figure 2:**
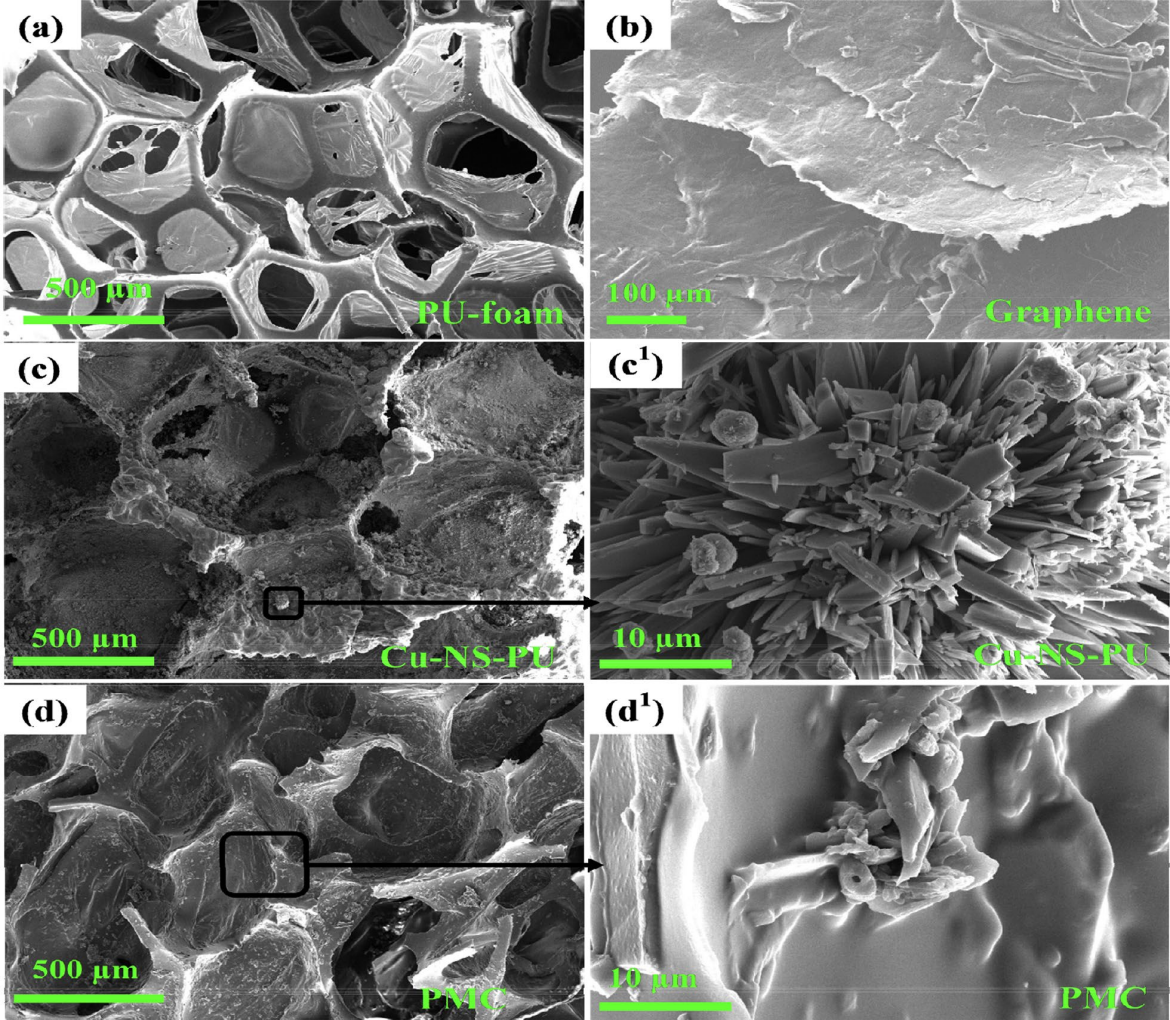
SEM images of (**a**) PU foam, (**b**) graphene nanosheet, (**c**,**c**') Cu-NS-PU, and (**d**,**d**') PMC.

Figure 2a shows the SEM images of PU foam. PU foam consists of several macropores. As observed from the SEM image, the size of pores varies from 150 to 500 µm that uniformly distributed over the entire foam. The interconnecting pores through a thin boundary create a framework. Figure 2b shows the nanosheet-like structure of mechanically exfoliated graphene. Multi-layer graphene can be seen from the images with sharp edges. Figure 2c–c’ shows images of Cu-NS-PU. As observed from the figure, Cu-NS uniformly deposited over the entire surface of PU foam. The magnified SEM image shows Cu-NS deposited onto the surface of PU foam. Figure 2d–d’ shows the SEM images of PMC samples, chitosan encapsulated on Cu-NS-PU can be observed from the images. The encapsulation of chitosan might decrease the Cu leach out from the PU surface. Also, it enhances the sorption ability of dyes and heavy metal ions as well as antibacterial activity^35,36^. Moreover, interconnecting pores might be beneficial for higher adsorption loading of contaminants, discuss later in the manuscript.

**Figure 3:**
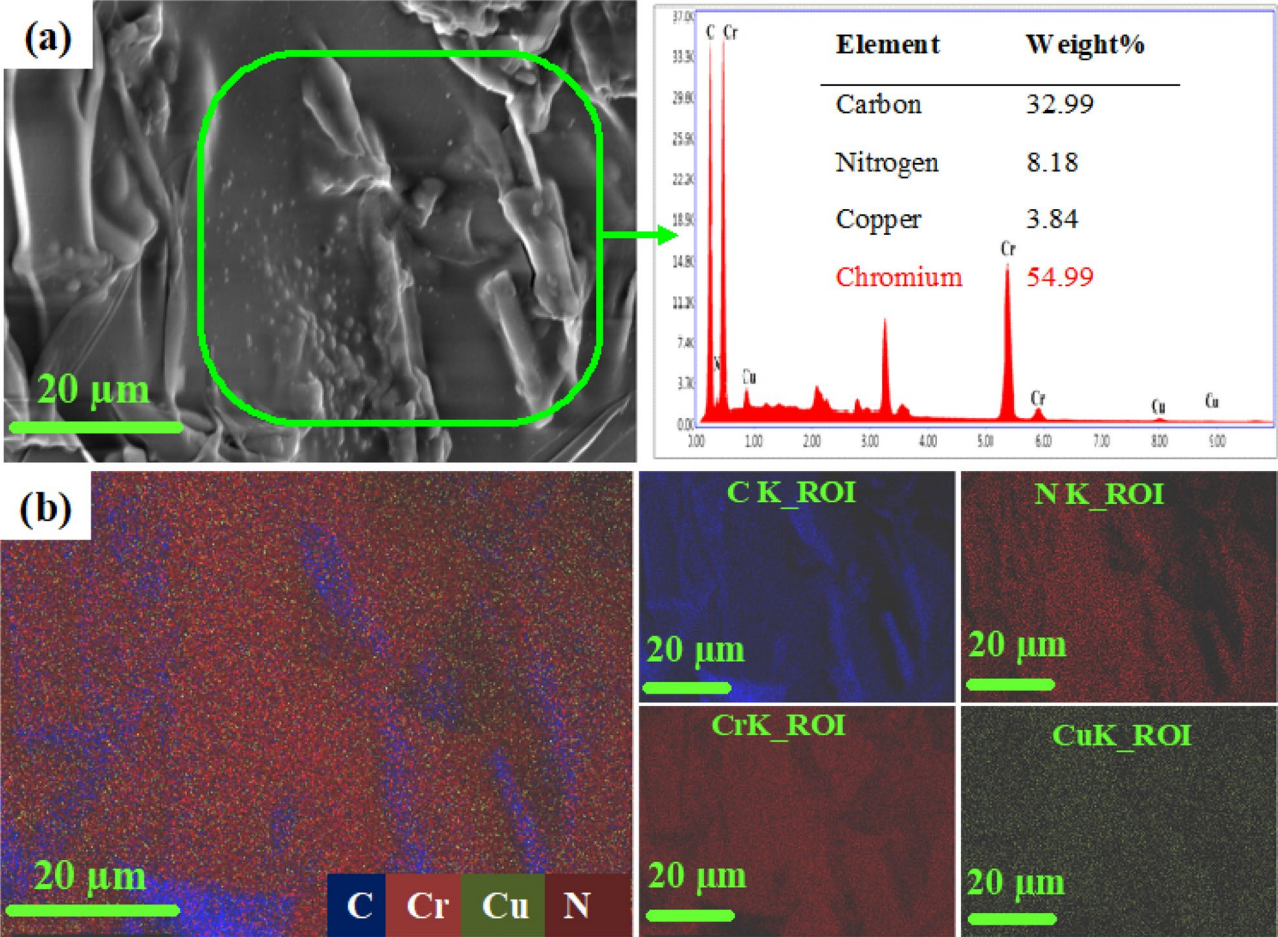
Elemental analysis of the Cr adsorbed PMC sample (**a**) elemental analysis, and (**b**) elemental mapping.

Figure 3a,b shows the elemental analysis and elemental mapping of the Cr(VI) adsorbed PMC samples. The presence of Cr content in the PMC samples attributed to the adsorption of Cr(VI) on the PMC sample. Moreover, approximately 55% of Cr (VI) indicated a higher sorption ability over the PMC based material.

### XRD analysis

**Figure 4:**
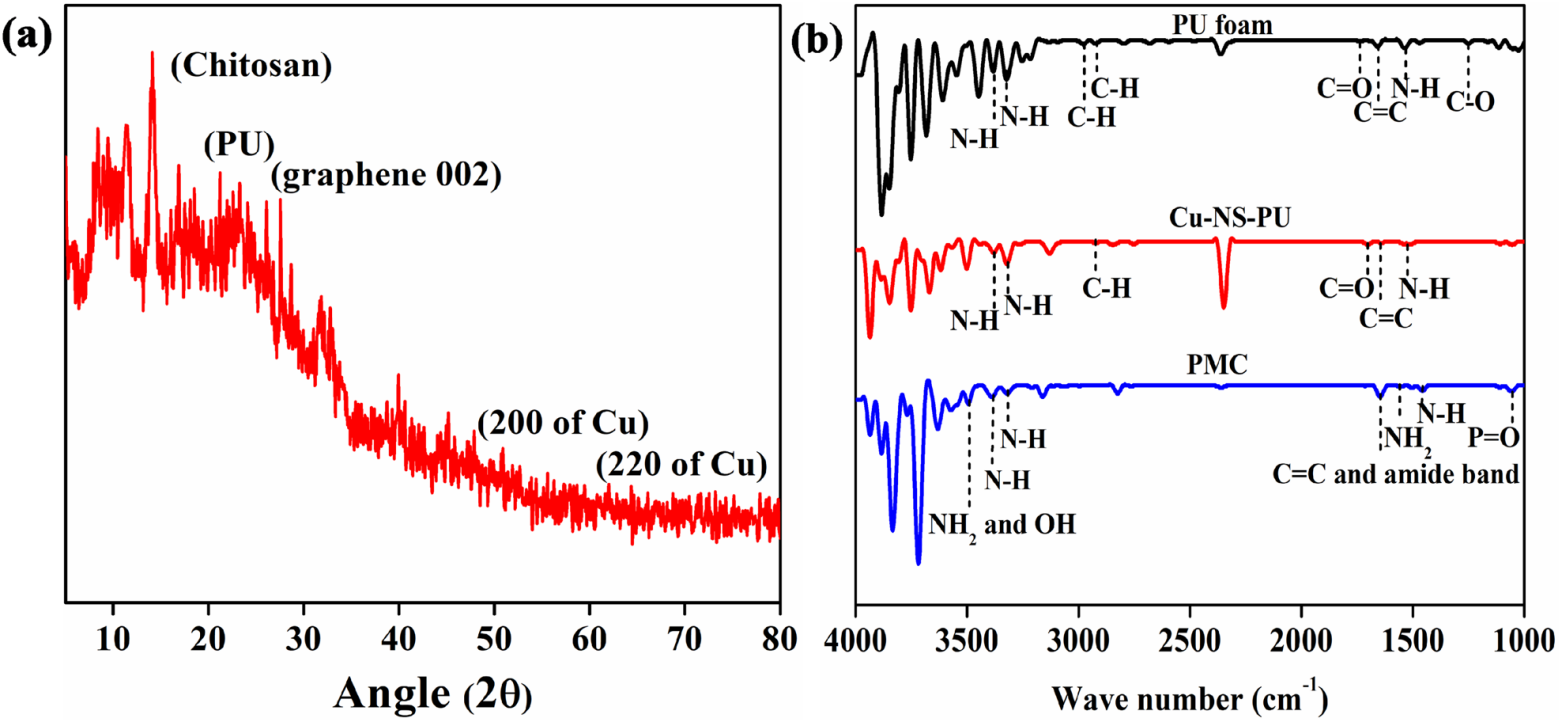
XRD analysis and FT-IR Spectra of the PMC material. (**a**) XRD analysis, (**b**) FT-IR Spectra.

The XRD diffraction pattern of Cu-NS-PU was shown in Fig. 4a. All the diffraction peaks were well matched with the solo PU, Chitosan, graphene, and Cu of Cu-NS-PU. As shown in Figure, Chitosan had a typical sharp peak at 10°. A broad peak appeared in the pattern at 19.25° and 23.28° was observed for the PU sponge. A characteristic peak of graphene was observed at 26°. The Cu diffraction pattern was observed at 2θ 45°, 50.6° and 74.9° which correspond to the crystallographic indices of the (111), (200) and (220), respectively, which proves that sample contained Cu in its pure metallic FCC phase (JCPDS No. 4-836)^37–39^. These results indicate that one-step loading of Cu-NS over PU was successfully done to develop Cu-NS-PU, which allows the function of graphene to be well dispersed and attached to the reticulated matrix of PU sponge to prepared PMC based adsorbent material.

### FT-IR analysis

Figure 4b shows the FT-IR spectra of the various constituents of the PMC based materials. The spectra are shown for the PU foam, Cu-NS-PU, and PMC material. The peaks observed at 1241, 1530, 1655, 1730, 2978, 2923, 3315, and 3382 cm^−1^ in the PU foam sample are assigned to –C–O, N–H, C=C, C=O, C–H, C–H, N–H, and N–H, respectively^40^. All characteristic peaks were observed in the Cu-NS-PU sample except 1241, 2978 cm^−1^. Moreover, the intensity of all characteristic peaks decreased in the Cu-NS-PU sample compare with that of the PU foam sample due to the Cu-NS deposition on the surface of PU foam^41^. All characteristics peaks were observed in PMC materials. Some new characteristics peaks at 1050, 1550, 1640, 1657, and 3490 cm^−1^ in the PMC sample are assigned to be P=O stretching of a phosphate group, NH_2_, amide band I, and NH_2_ and OH group, respectively that belongs to chitosan^42^. The FT-IR spectra confirm that the PMC material was successfully synthesized. These functional groups aided advantageous to adsorb chemical contaminants as well as remove biological contaminants.

### Adsorption study

**Figure 5:**
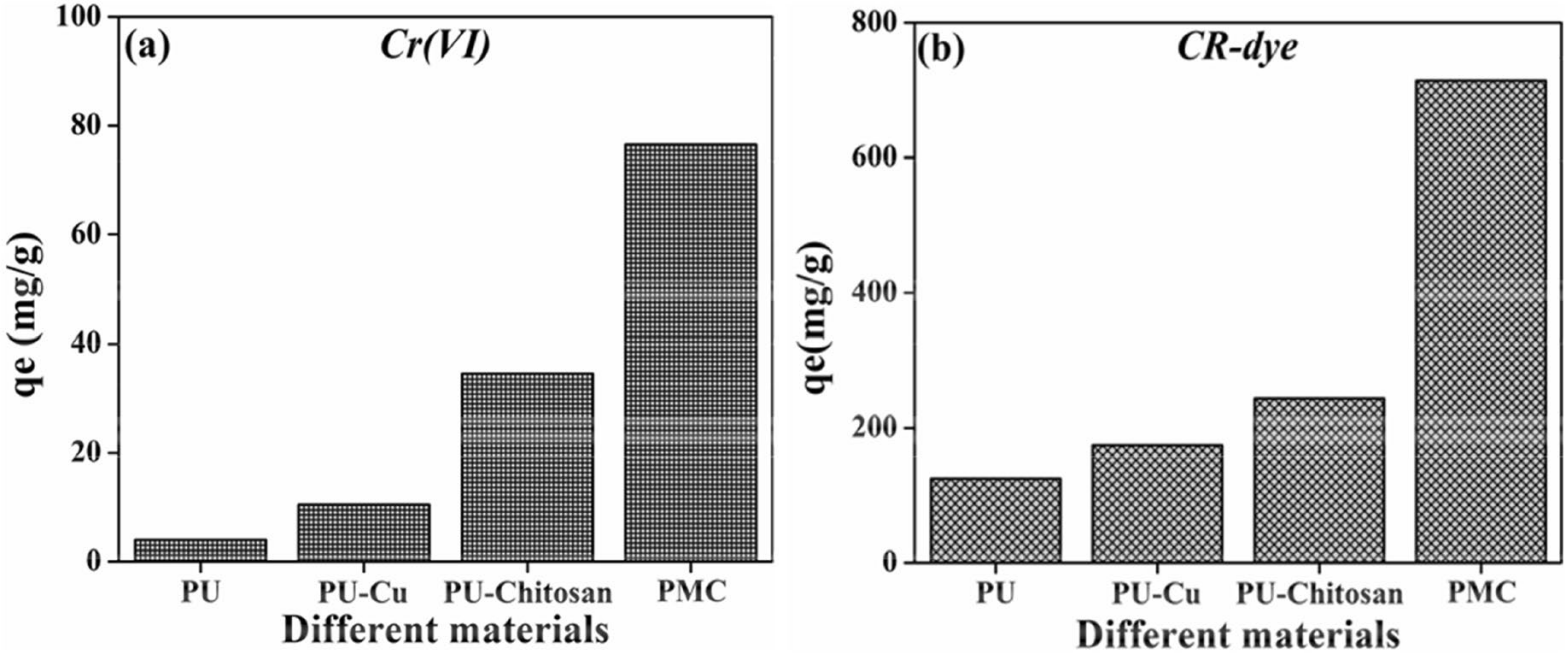
Different PU based composite materials for the adsorption of Cr(VI), and CR-dye.

Figure 5 shows the different PU-based composite materials for the adsorption of Cr(VI) and CR-dye. Figure 5a shows the adsorption of PU-based composite materials against Cr(VI) ions. As observed from the figure, PU has minimum sorption ability. The deposition of Cu-NS onto the PU surface increases the sorption ability of the Cr (VI) ions. Pu-chitosan-based composite has a higher sorption ability compare with that of PU, and PU-Cu-NS due to positively charged surface and its surface functional group. Interestingly, the incorporation of Cu-NS, chitosan, graphene within the PU surface (PMC) materials shows the superior sorption ability. Figure 5b shows the adsorption of PU-based composite materials against CR-dye molecules. A similar trend like adsorption of Cr (VI) ions was also observed in CR-dye. The data suggested that PMC frameworks show higher sorption ability against both contaminants. Therefore, PMC frameworks might be used for the further analysis.

#### Kinetics study

**Figure 6:**
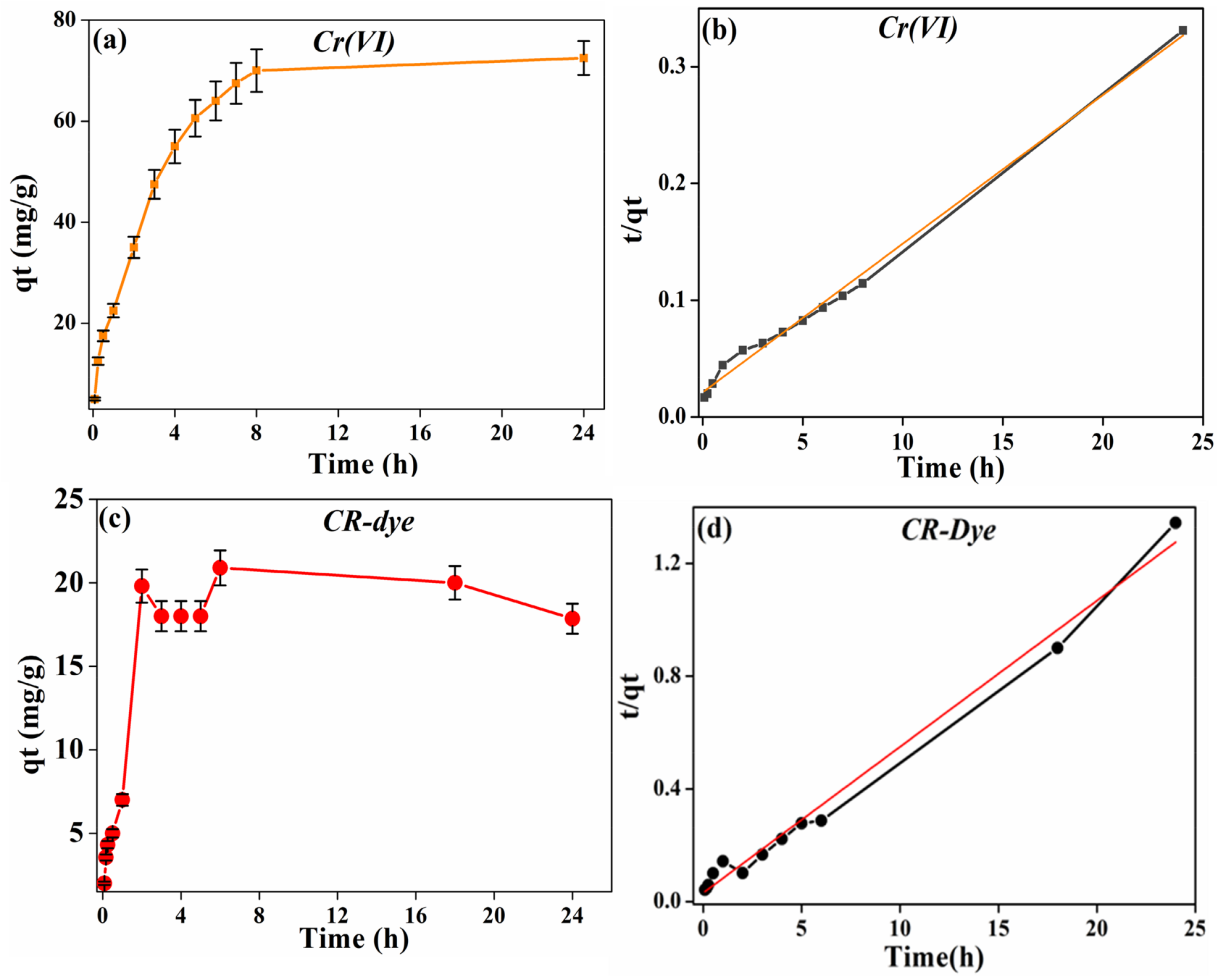
Adsorption kinetics of Cr(VI), and CR-dye on the prepared PMC based material. (**a**) Time study of Cr(VI), (**b**) pseudo-second-order kinetic model of Cr(VI), (**c**) time study of CR-Dye, and (**d**) pseudo-second-order kinetic model of CR-Dye.

Kinetics experiment was performed to study the rate of adsorption of Cr (VI) and CR-dye, at 150 and 10 ppm, respectively, in the time range of 10–1440 min. Figure 6 shows the adsorption kinetics of Cr(VI) and CR-dye on the prepared PMC based material. As observed from the Figure, the adsorption of Cr (VI) and CR-dye on PMC material was rapid during the initial 8 h, after approaching the equilibrium concentration (24 h), the rate slowed down. Pseudo-second-order kinetic models were used to explain the uptake of Cr(VI) and CR dye with time. The model equation was explained as follows:

2 $$\frac{1}{kqe}+\frac{t}{qe}=\frac{t}{qt}$$

where qe is the equilibrium adsorption loading, and qt is the adsorption capacity (loading) at time t and k is the rate constant. Figure 6, and Table 1, describes the kinetic model and respective parameters as per Eq. (2) fitted to the data. The numerical value of the rate constant k calculated from the intercept of the line, respectively, was 0.0076 and 0.087 g/mg h^−1^ for Cr(VI) and CR dye, respectively. The relatively higher R^2^ value (0.9942 and 0.9889) validates the applicability of the pseudo-second-order rate kinetics to the adsorption of Cr (VI) and CR dye on the PMC materials.

**Table 1:** Psuedosecond-order kinetic model constant for Cr (VI) and CR dye.

Analyte	Pseudo second order	
	R^2^	K_2_′ (g mg^−1^ h^−1^)
Cr ion	0.9942	0.0076
Congo red dye	0.9889	0.087

#### Equilibrium study and adsorption isotherms

**Figure 7:**
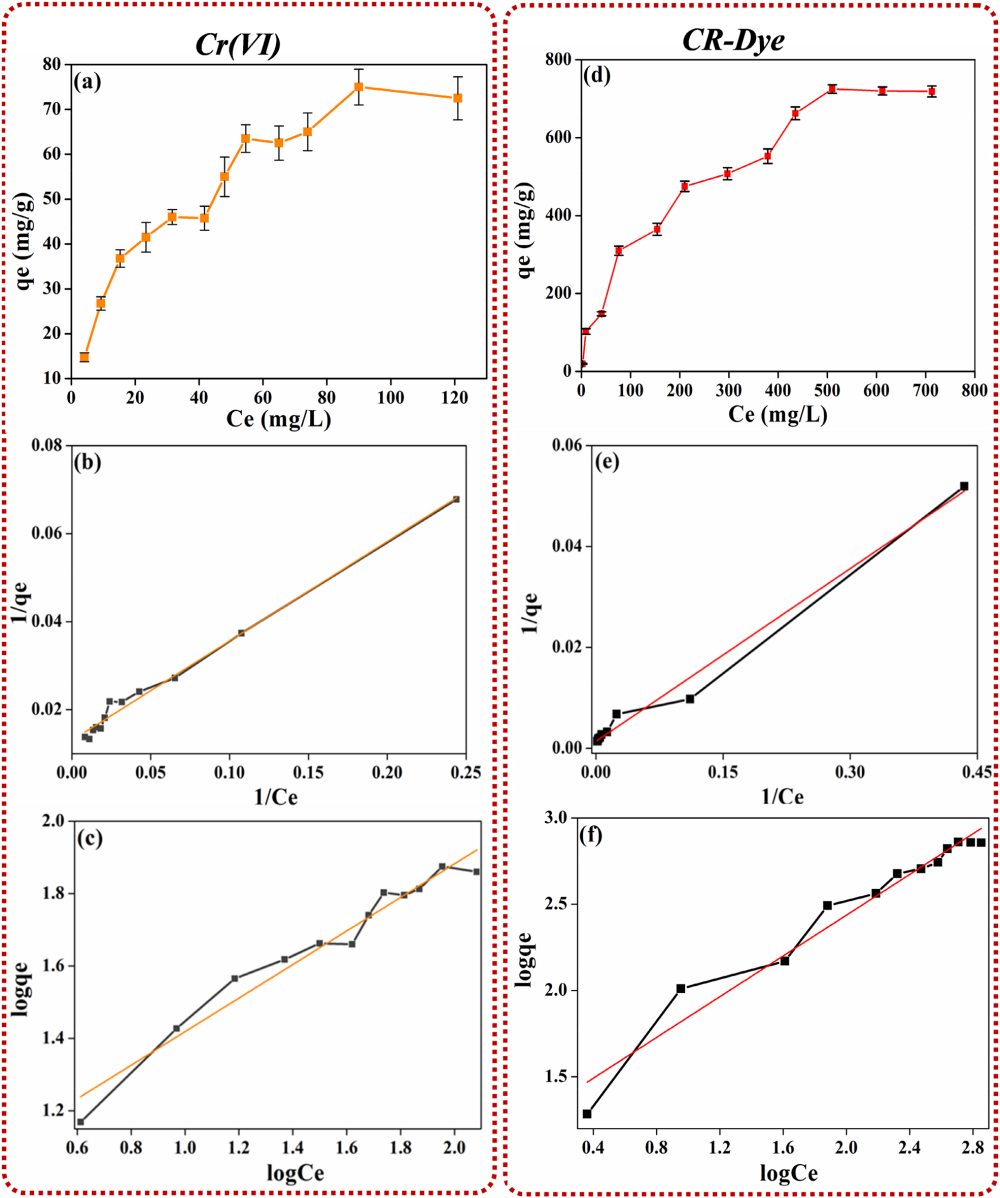
Effect of liquid phase concentration of (**a**–**c**) Cr(VI) adsorption (**d**–**f**) CR-dye adsorption. (**a**) Adsorption Isotherm, (**b**) Langmuir isotherm, (**c**) Freundlich isotherm, (**d**) Adsorption isotherm, (**e**) Langmuir isotherm, and (**f**) Freundlich isotherm.

Figure 7 shows the solid phase equilibrium concentrations of Cr(VI) and CR-dye for different aqueous phase concentrations. The adsorption capacity of the PMC material for Cr(VI) and CR was determined at 30 °C in the aqueous phase concentration range of 10–150 and 10–800 mg L^−1^ respectively. The adsorption capacity reached equilibrium with increasing aqueous phase concentrations. The Langmuir and Freundlich isotherm was used in the linearized form to fit the equilibrium data:

3 $$qe=\frac{qmaxKCe}{1+KCe}$$

4 $$Qe=Kf{Ce}^{1/n}$$

where qe is the solute loading (mg g^−1^) at equilibrium, qmax is the maximum adsorption loading (mg g^−1^), Ce is the equilibrium concentration (mg L^−1^) and K is the Langmuir constant. The Langmuir model assumes monolayer coverage of adsorbed molecule over adsorbent however Freundlich model assumes adsorbed molecules have heterogeneous adsorption sites and the adsorbed molecules or ions have a mutual effect on each other. As shown in Fig. 7, the linearized form of the Langmuir equation fits the data for PMC material reasonably well, indicating the monolayer surface coverage of the Cr(VI) and CR on PMC material. The maximum adsorption for Cr(VI) and CR were 76.9 and 714 mg g^−1^ calculated from Langmuir isotherm after 24 h of equilibrium at 150 mg L^−1^ and 800 mg L^−1^ of initial concentration showing the applicability of PMC material for Cr(VI) and CR-dye. Table 2 lists the numerical values of the Langmuir and Freundlich adsorption constant and the regression correlation coefficient. The values of K_L_ range within 0 to 1, which demonstrates that the samples have a better absorption for Cr(VI) ions and CR-dye.

**Table 2:** Langmuir and Freundlich isotherm model parameters for Congo red dye and Cr(VI) at 30 °C.

Adsorbate	Adsorbent	Langmuir isotherm			Freundlich isotherm		
		q_max_(mg/g)	b (Lmg^−1^)	R^2^	K_F_ (Lg^−1^)	n_F_	R^2^
CR	PMC	714.3	0.01226	0.9881	16.98	1.64	0.9589
Cr (VI)		76.9	0.0575	0.9901	9.01	2.155	0.961

#### Effect of temperature and thermodynamics of Cr(VI) and CR adsorption

**Figure 8:**
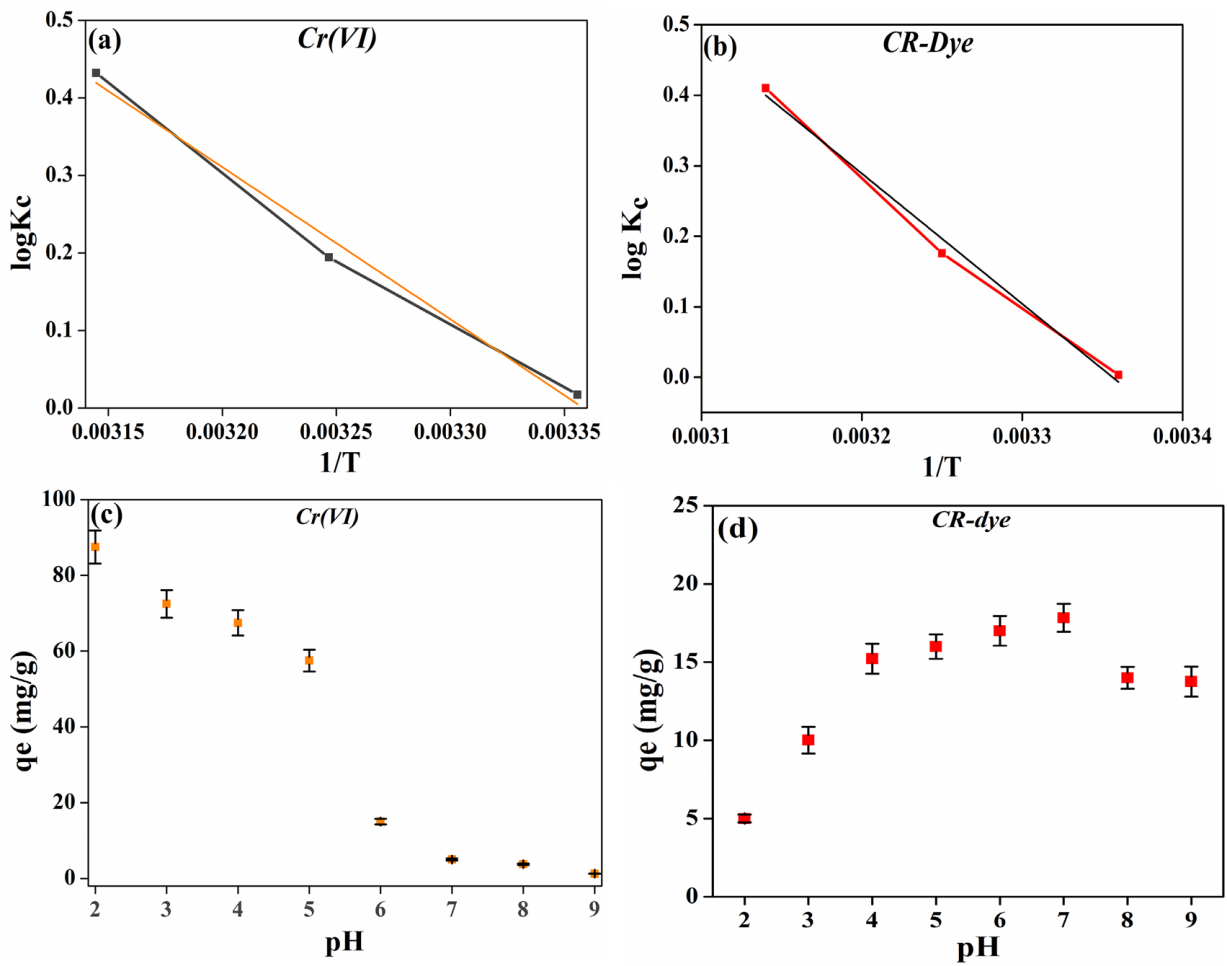
**T**hermodynamics and pH effects on adsorption. (**a**,**c**) Cr(VI), and (**b**,**d**) CR-dye.

Effect of temperature on Cr(VI) and CR-dye adsorption on PMC material was studied at three different temperatures viz., 298, 308, and 318 K with keeping rest parameters constant. Figure 8a,b describes the effect of temperature on adsorption. It can be observed from the figure that the maximum adsorption capacity decreased with increasing adsorption temperature, indicating the adsorption to be exothermic. Table 3 shows the calculated values of different parameters from the Langmuir isotherm equation at 298, 308, and 318 K.

**Table 3:** Thermodynamic parameters for adsorption of Cr(VI) and CR-dye on the PMC material.

Analyte	T(K)	Kc	ΔG (kJ mol^−1^)	ΔH (kJ mol^−1^)
Cr ion	298	1.040816	− 0.0991	− 37.56
	308	1.564103	− 1.145	
	318	2.703704	− 2.629	
CR dye	298	1.01	− 0.02	− 35.4
	308	1.5	− 1.04	
	318	2.57	− 2.5	

Calculations were performed to extract the thermodynamic parameters from the batch data. It was also necessary to conclude whether the adsorption process was exothermic and spontaneous. The thermodynamic parameters, namely Gibb's free energy change (ΔG°), enthalpy change (ΔH°), and entropy changes (ΔS°), were calculated using the following equations:

5 $$-RTlnK=\Delta$$

6 $${\Delta H}^{o}-T\mathrm{\Delta S}^\circ =\mathrm{\Delta G}^\circ $$

7 $$\frac{\mathrm{\Delta H}^\circ }{RT}\times \frac{\mathrm{\Delta S}^\circ }{R}=lnK$$

where R is the gas constant, and T is the temperature (K). ΔG° was calculated from the K obtained from the Langmuir equation. Table 3 lists the numerical values of ΔG° obtained for different temperatures for Cr(VI) and CR.

The negative value of ΔG° describes the spontaneous nature of adsorption for both Cr(VI) and CR dye. On increasing the temperature, ΔG° decreased, indicating that a relatively lower temperature was unfavorable for adsorption. Figure 8a,b shows the plot of lnK vs. 1/T and is found to be linear. ΔH° was calculated to be − 37.56 and − 35.4 kJ mole^−1^ from the slope of the line (Eq. (7)) for Cr(VI) and CR dye, confirming the adsorption of Cr(VI) was exothermic respectively.

#### Effect of pH on Cr(VI) and CR dye adsorption

Figure 8c,d shows the effect of pH on adsorption of Cr(VI) and CR dye on PMC based adsorbent material. Figure 8c it is visible that on changing the pH value from 2 to 9, adsorption of Cr(VI) was significantly affected and the highest adsorption loading (87.5 mg g^−1^) was observed at pH ~ 2. The reason for this adsorption capacity is thought to be that the adsorption sites occupy the anionic species, such as HCrO_4_^−^, CrO_4_^−2^, and Cr_2_O_7_^−2^, these anionic form of Cr(VI) mainly found in acidic pH. Next, PMC consist of several amine group, at acidic pH these react with proton to form –NH_3_^+^, groups which promotes Cr(VI) anion to move towards adsorbent and create electrostatic attraction.

Surface charges of PU, chitosan, and graphene have been reported in several literatures. According to them, the surface charge of PU, and Chitosan is positive^43,44^. However the graphene surface is negative^45^. Next, while synthesizing composite the overall surface charge is positive due to presence of several positive moieties. The reason behind the positive surface of PMC at the acidic pH ranging from 1.0 to 4.0, is availability of adequate H^+^. Therefore, the amine groups from the chitosan moiety of the adsorbent were easily protonated and positively charged, which promoted the approach of negatively charged Cr(VI) species (HCrO_4_^−^) attributed to the electrostatic interaction^46^. However, basic pH exhibits the opposite mechanism as repulsion was the predominant force to expel Cr(VI) far away from the adsorbent surface subsequently low adsorption capacity^47^.

Similarly, the effect of pH on adsorption of CR dye was shown in Fig. 8d. The maximum loading was observed at pH 7 (17.84 mg g^−1^) in 10 mg L^−1^ of CR concentration. Adsorption efficiency changes with changing pH value. As at acidic pH, a large H^+^ concentration results in protonation of the several group such as amine with PMC framework, give rise to electrostatic interactions^48^. However at neutral pH, protonation of amine groups was nominal due to the negligible concentration of free H^+^. Next, CR is an acidic dye in neutral condition. It exists in the anionic form in solution. Additionally, in PMC the surface of aminated graphene (chitosan encapsulated graphene) is positive. It can be expected that the total surface charge over the PMC probably positive during this stage, (as mentioned above) which gives rise to electrostatic attraction. Therefore, adsorption ability is higher at this pH. However, the acidic pH of dye solution gives rise to repulsion between adsorbent and adsorbate surface due to similar polarity over CR molecule and surface of PMC. On further increasing the pH above 7.0, excessive OH ions compete with the sulfonate groups of CR and the active site for dye adsorption is reduced compared to neutral pH which decreases the overall adsorption of CR dye onto PMC based adsorbent material^49^. Therefore, the prepared PMC based adsorbent materials effectively remove both contaminants (Cr(VI) and CR-dye) from water.

#### Recyclability of PMC framework

**Figure 9:**
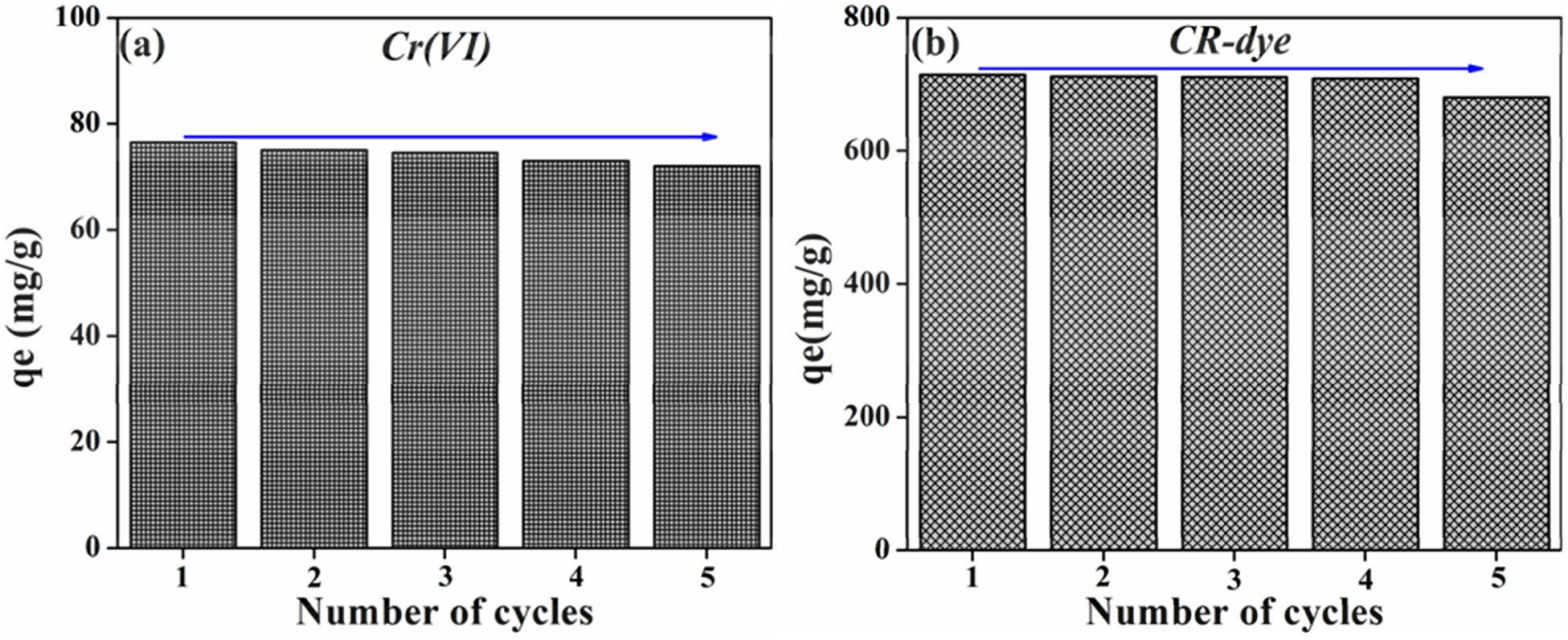
Cycling stability of PMC materials against (**a**) Cr(VI), and (**b**) CR-dye.

Recycling of the materials is an important factor to determine the efficacy of the designed material. Usually, ideal adsorbent materials should be stable after various adsorbent runs. The recyclability test assay of the PMC framework was performed against Cr(VI) and CR-dye using a simple washing procedure. After each adsorption cycle, the PMC framework was washed several times using ethanol: water (1:4) ratio to remove metal ions and dye molecules from the surface of PMC and then dry at 60 °C for 24 h. Figure 9 shows the recyclability test assay of the PMC framework against Cr(VI) and CR-dye contaminants. As observed from the figure, insignificant changes were observed up to five consecutive cycles. The re-usability ensures the high stability during adsorption. Therefore, the prepared PMC frameworks remain stable throughout the adsorption experiments and show extra-ordinary re-usability, which is a chief factor for end applications.

### Antibacterial activity

**Figure 10:**
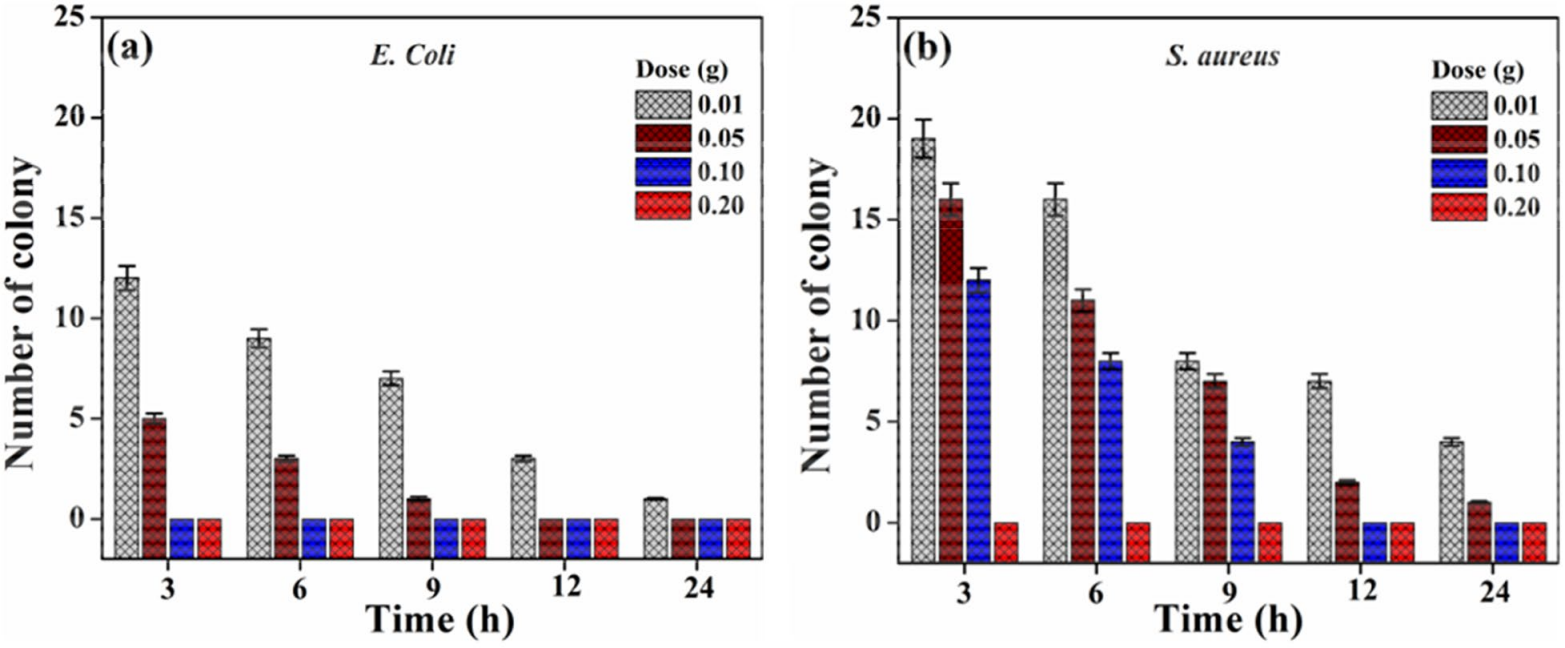
Antibacterial activity of PMC materials against (**a**) *E. Coli*, and (**b**) *S. aureus.*

The antimicrobial behavior of different weights (0.01 to 0.20 g) of PMC materials was tested against both Gram-negative (*E. Coli*) and positive (*S.aureus*) bacterial strains with different exposure times (3–24 h). Figure 10a,b show the antibacterial analysis of PMC against both *E. Coli* and *S.aureus,* respectively. There were numerous significant observations. (1) PMC materials had suppression or inhibitory effects against both *E. Coli* and *S.aureus*bacterial strains. (2) PMC materials have comparatively higher performance against *S.aureus* bacterial strain. (3) The concentration at or below 0.10 g of the PMC sample, the *E. Coli* and *S.aureus* bacterial colonies were suppressed or inhibited for lesser exposure time (3 h), and 12 h, respectively, and (4) the higher concentration at 0.20 g was sufficient to kill or inhibit both bacterial strains at 3 h.

Table 4 show the comparative data of the different hybrid materials used for the removal of contaminants from water. The data suggested that the prepared materials have superior adsorption loading among all of them. Moreover, most of the materials can remove either chemical or biological contaminants from water. In this context, the prepared PMC based adsorbent materials or filter has the potential ability to remove both chemical and biological contaminants from water, thereby PMC based adsorbent materials or filter efficiently used for the treatment of water.

**Table 4:** Comparison of different hybrid materials used for the removal of contaminants.

S. No	Adsorbent	Contaminants	C(mg/l)	qmax(mg/g)	References
1	PMC	Cr(VI) CR-dye *E. Coli* *S.aureus*	10–150 10–1000 10–4 CFU/ml 10–4 CFU/ml	~ 76.9 ~ 712	This study
2	Fe PhB A CNF	Cr(VI)	10–150	45	^1^
3	Graphene oxide-cyclodextrin chitosan	Cr(VI)	5–50	67	^39^
4	PU-Cu NPs	*E. Coli* *S.aureus* *K. marxianus*	–	Inhibit the growth	^40^
5	PU-keratin-AgNP-lodoacetic acid	*E. Coli* *S. aureus*	–	Inhibit the growth	^41^
6	Zr-MOF-PU foam	CR-dye	5–30	19.57	^42^
7	Chitosan-Silica-aerogel	CR-Dye	–	150	^43^
8	Cellulose nanofibril-carbon based aerogel	CR-Dye	–	585.3	^44^

## Conclusion

The PMC framework has synthesized using the CBD process and applied as complete decontamination including chemical and biological contaminants from wastewater. The synthesized framework exhibits excellent adsorption towards Cr(VI) and CR-dye as chemical contaminants and *E. coli* and *S. aureus* as biological contaminates. The maximum adsorption capacity was found to be 76.5 mg g^−1^ and 714 mg g^−1^ for Cr(VI) and CR-dye, respectively. Pseudo-second-order kinetic model was the best-demonstrated model on the adsorption process. Langmuir and Freundlich's isotherms were the best-fitted models for Cr(VI) and CR adsorption on the PMC framework. pH plays a significant role in the adsorption process as acidic pH was best for Cr(VI) adsorption while neutral pH was the best pH for CR adsorption. Surface charge plays important role in the adsorption process as postulated mechanism proves the electrostatic attraction due to adsorbent charges. The as-prepared hierarchical PMC framework is promising adsorbents for the removal of both biological and chemical contaminants from wastewater because of high surface charge, simple synthesis process, and excellent efficiency towards contaminants.
